# The Use of Augmented Reality Technology in Medical Specimen Museum Tours

**DOI:** 10.1002/ase.1822

**Published:** 2018-11-19

**Authors:** Atsushi Sugiura, Toshihiro Kitama, Masahiro Toyoura, Xiaoyang Mao

**Affiliations:** ^1^ Center for Life Science Research University of Yamanashi Chuo Yamanashi Japan; ^2^ Interdisciplinary Graduate School University of Yamanashi Kofu Yamanashi Japan

**Keywords:** gross anatomy education, medical education, anatomy museums, specimen museums, augmented reality, gesture recognition

## Abstract

Human anatomical specimen museums are commonly used by medical, nursing, and paramedical students. Through dissection and prosection, the specimens housed in these museums allow students to appreciate the complex relationships of organs and structures in more detail than textbooks could provide. However, it may be difficult for students, particularly novices, to identify the various parts of these anatomical structures without additional explanations from a docent or supplemental illustrations. Recently, augmented reality (AR) has been used in many museum exhibits to display virtual objects in videos captured from the real world. This technology can significantly enhance the learning experience. In this study, three AR‐based support systems for tours in medical specimen museums were developed, and their usability and effectiveness for learning were examined. The first system was constructed using an AR marker. This system could display virtual label information for specimens by capturing AR markers using a tablet camera. Individual AR markers were required for all specimens, but their presence in and on the prosected specimens could also be obtrusive. The second system was developed to set the specimen image itself as an image marker, as most specimens were displayed in cross section. Visitors could then obtain the label information presented by AR without any markers intruding on the display or anatomical specimens. The third system was comprised of a head‐mounted display combined with a natural click interface. The system could provide visitors with an environment for the natural manipulation of virtual objects with future scalability.

## Introduction

Medical museums exhibiting anatomical and pathological specimens first appeared in the 16th century (Cole, 1949; Bates, 2008). Many medical museums, as exemplified by the Hunterian Museum belonging to the Royal College of Surgeons in England (Turk, 1994), were founded in Europe. Since the early 19th century, these museums have played an important role in medical education (Marreez et al., 2010). There are numerous outstanding examples of their contribution to modern medical education (Shibata et al., 1991; Wakefield, 2007; Marreez et al., 2010; Diaz‐Perez et al., 2014; Moro et al., 2017). Recently, however, some of these institutions appear to have shrunk or scaled back their exhibitions. This has occurred due to several reasons, such as universities’ increased need for laboratory and teaching space, as well as difficulty in updating and maintaining the exhibitions (Fulcheri, 1996; Ferrari et al., 2001; Wakefield, 2007; Küçük et al., 2016).

At the same time, numerous developments in information and communication technology have significantly influenced medical education. Medical educators have experimented with many recent developments, such as three‐dimensional (3D) printing (Preece et al., 2013; McMenamin et al., 2014; Watson, 2014; Jones et al., 2016; Yao et al., 2014), audiovisual studies (Pavese et al., 2012; Rössler et al., 2012; Benninger et al., 2014; Blake, 2014; Dash et al., 2015; Rinaldi et al., 2017), and computer‐generated imagery including virtual reality (VR) combined with a mobile device (Luursema et al., 2006; McGhee, 2010; Engelke et al., 2013; Liu et al., 2013; Kockro et al., 2015; Miki et al., 2016; Yeo et al., 2011). In particular, these modalities have been applied to anatomical and pathological education, especially with regards to understanding the 3D structure of the body in physiologic and pathologic states. Such technologies may also bridge many of the difficulties in the upkeep and use of medical museums in medical education (Marreez et al., 2010).

### Augmented Reality Technology

Augmented reality (AR) is a technology that enables the display of virtual objects in the real world by overlaying supplementary information on the real environment. In order to smoothly generate AR environments, the integration of spatial and temporal information in virtual and real environments is critical. Two methods are available for spatial information integration: the sensor‐based method and the image marker‐based method. The sensor‐based method identifies a position with high accuracy in the real environment using a sensor, such as a global positioning system (GPS) or an acceleration sensor. Augmented reality applications using handheld GPS devices have been previously utilized for field workers (Odashima et al., 2003; Kamat and El‐Tawil, 2007; Schall et al., 2009). Another recent example of a GPS‐based AR is the mobile game “Pokémon Go” (Niantic Inc., San Francisco, CA), in which players used their mobile devices to locate and capture virtual creatures, called Pokémon, in real surroundings. Employing cameras and gyroscopes on players’ mobile devices, AR technology superimposed these creatures on the real backgrounds captured by the players’ camera. Although the game promoted outdoor physical activity and popularized GPS AR technology, it received both positive (Althoff et al., 2016; Nigg et al., 2017; Xian et al., 2017) and negative critiques (Ayers et al., 2016; Serino et al., 2016) for its effects on players and public health (Wagner‐Greene et al., 2017).

Despite the effectiveness of position detection in outdoor areas, this modality is limited for indoor activities due to poor GPS signal reception inside buildings. Bombara et al. (2003) proposed a guidance system for museum tours that would employ infrared radiation sensors to specify a visitor’s location. Although the system appeared to be effective, the cost of sensor installation would be a major limitation. Augmented reality systems using wireless acceleration sensors combined with image processing can enable object detection when an item is picked up and viewed by a user, even among many surrounding objects (Karatsu et al., 2010).

**Figure 1:**
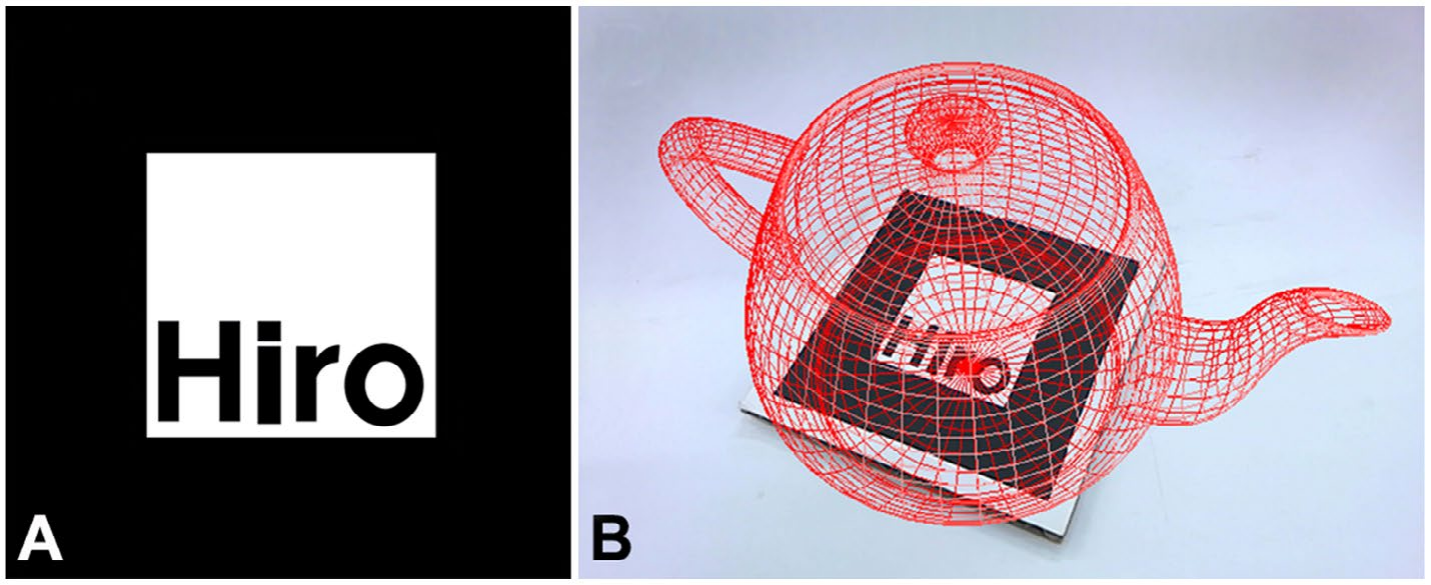
Augmented reality (AR) marker used in ARToolKit (version 2) and an example of a displayed virtual object. A, Example of an AR marker that has a square bold frame and a pattern in the center to distinguish it. The pattern information of the AR marker is pre‐registered in the system. A user captures the AR marker with the device camera; B, Virtual object displayed on the AR marker when the captured image is satisfactorily detected by the system.

In the image marker method, each marker typically has a distinguishing pattern created from bold, square frames (Fig. 1A) for easy recognition by the camera of a user’s device. The real and virtual environments are integrated by aligning the center of the AR marker with that of the coordinates in the virtual environment, thereby detecting the orientation of the marker to present a virtual object on it as a synthesized image (Fig. 1B).

### Augmented Reality in Education

The educational potential of AR technology is most applicable in demonstrating the spatial relationships of elements within a 3D space (Kerawalla et al., 2006; Cheng and Tsai, 2013; Wu et al., 2013), and may thus serve as an interactive learning tool for various fields of study (Dunleavy et al., 2009; Andujar et al., 2011; Bujak et al., 2013; Chiang et al., 2014; Küçük et al., 2013). A variety of computer‐based training systems have been developed for anatomy education, such as the freely available BioDigital Human three‐dimensional computer graphics (3DCG) software (BioDigital, 2017) and the Human Anatomy Atlas smartphone application software by Visible Body^®^ (Visible Body, 2017). This software allows users to engage with content, learn independently, and explore images of 3D anatomical structures. AR technology combined with 3DCG images can also be applied to anatomy education (Ferrer‐Torregrosa et al., 2015; Küçük et al., 2016; Moro et al., 2017), as well as other interactive learning techniques (Chien et al., 2010), overlaid images using a magic mirror (Meng et al., 2013), or even haptic feedback systems (Sakellariou et al., 2009; Luciano et al., 2011; Yeom, 2011). Each system was constructed using AR technology to enable 3D structural understanding or surgical skill training. Previous studies have suggested that such systems not only enhance learners’ motivation, but also their capacity for independent learning.

Various types of AR technology have also been developed for museums to enhance the visitor’s experience: mobile vision‐based AR systems can enable the presentation of contextual content in front of artworks (Tilon et al., 2011), as well as monuments, sculptures, or architecture (Keil et al., 2013); AR technology can present a combined system of virtual museums using a web browser (Wojciechowski et al., 2004); and AR technology can be used with a digital video projection system in a science museum (Yoon et al., 2012). The effect of using AR guides on visitor activities was also examined in an art museum under various conditions, including using a mobile AR guide system, an audio guide, or no guide (Chang et al., 2014). The results demonstrated that providing visitors with an AR guide clearly facilitated effective learning (i.e., changes in tour behavior such as flow experience and extending the time spent focusing on paintings). These examples illustrate the unique applications of AR systems in museum exhibitions, and how they can enhance visitors’ learning experience in an enjoyable fashion.

### Information Communication and Computing Technology Applied to Medical Museums

In contrast to the circumstances surrounding traditional anatomy/pathology museums described earlier, VR technology and other informatics tools are about to revolutionize such museums by serving as a new educational tool (Wakefield, 2007; Marreez et al., 2010; Diaz‐Perez et al., 2014). In these examples, the medical museum is typically engaged in the anatomy and pathology curriculum for medical students. The use of an integrated curriculum with informatics tools enhanced students’ learning experiences and resulted in better exam scores compared with traditional lectures. However, despite these examples, the application of AR technology in anatomy and pathology museum exhibits has so far not been investigated.

The specimen museum at the University of Yamanashi, Faculty of Medicine (UYFM) is relatively smaller than some of the prominent museums cited earlier. The authors manage the museum and act as docents to present exhibitions to visitors (i.e., students of medical, nursing, or paramedic courses at the university and from the surrounding areas). Medical students in the UYFM can visit the museum at any time during school hours, although only a small number of students actually do visit, probably because it is not included in the curriculum requirements for medical students, or because students are unaware of its presence. These students attend numerous lectures and engage in various activities, such as anatomy and pathology laboratory time (i.e., cadaveric dissection and microscopy). However, the number of total hours of basic biomedical science training has been decreasing in medical curricula, including that of gross anatomy teaching, along with an increase in case‐based learning relevant to health and disease in the form of either group interactive sessions or active learning (Drake et al., 2009). Therefore, improving the traditional medical specimen museum descriptions may be necessary to provide a more effective or attractive learning environment in accordance with these changing trends. Conversely, nursing and paramedical students learn anatomy and pathology only through a limited number of lectures, textbooks with illustrations, and video material. Therefore, touring a dissection session for medical students and visiting a medical specimen museum are invaluable for these nursing and paramedical students. This experience‐based learning with explanations offered by a docent can help promote the conceptual understanding of macroscopic structures and their relations to the functions of various systems. However, in scenarios in which students browse at their own pace, exhibition description panels are unable to satisfactorily fulfill the purpose of the exhibits. Innovative improvement for an attractive exhibition description, which may also be applicable to self‐learning or active learning, must be a key element for modern students. Whereas, AR technology seems to have potential in medical education, as shown in the studies cited above, only a limited number of studies have been conducted on medical museums. Therefore, this research aims to enhance the medical museum for more frequent usage and to make it more effective as a learning support environment, especially through self‐directed learning using AR technology.

## Materials and Methods

**Figure 2:**
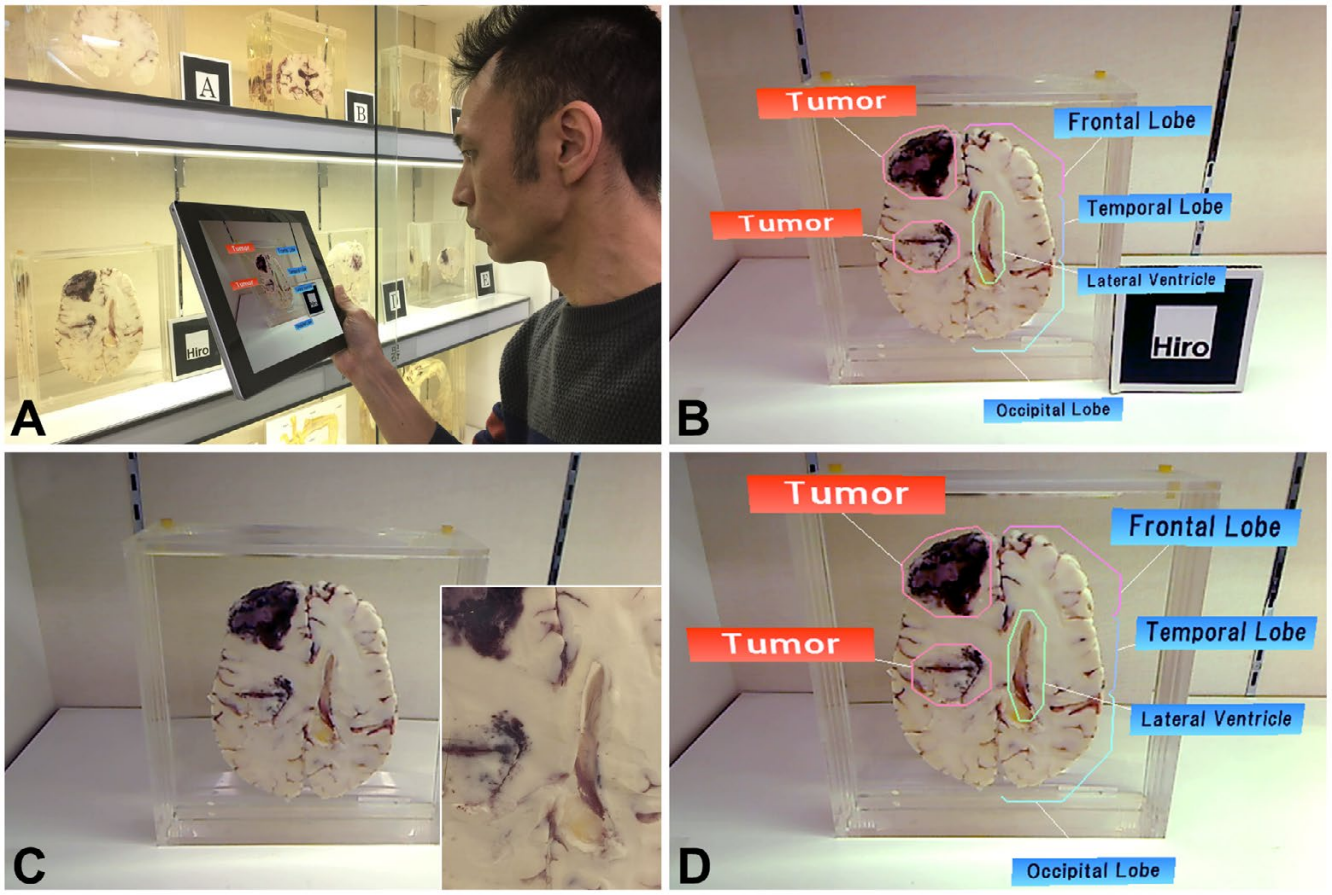
Two developed systems with different augmented reality (AR) tracking techniques (A and B, AR marker system; C and D, the image marker system). A, A user capturing the AR marker with the tablet’s camera; B, Virtual part labels displayed on the specimen when the system detects the AR marker; C, An overall cross‐section image. A rectangular part of the cross‐section image clipped as the marker (inset); D, Virtual part labels displayed on the specimen when the system detects the captured image as the marker. Note that the system allows the user to obtain the AR information without being aware of the marker.

**Figure 3:**
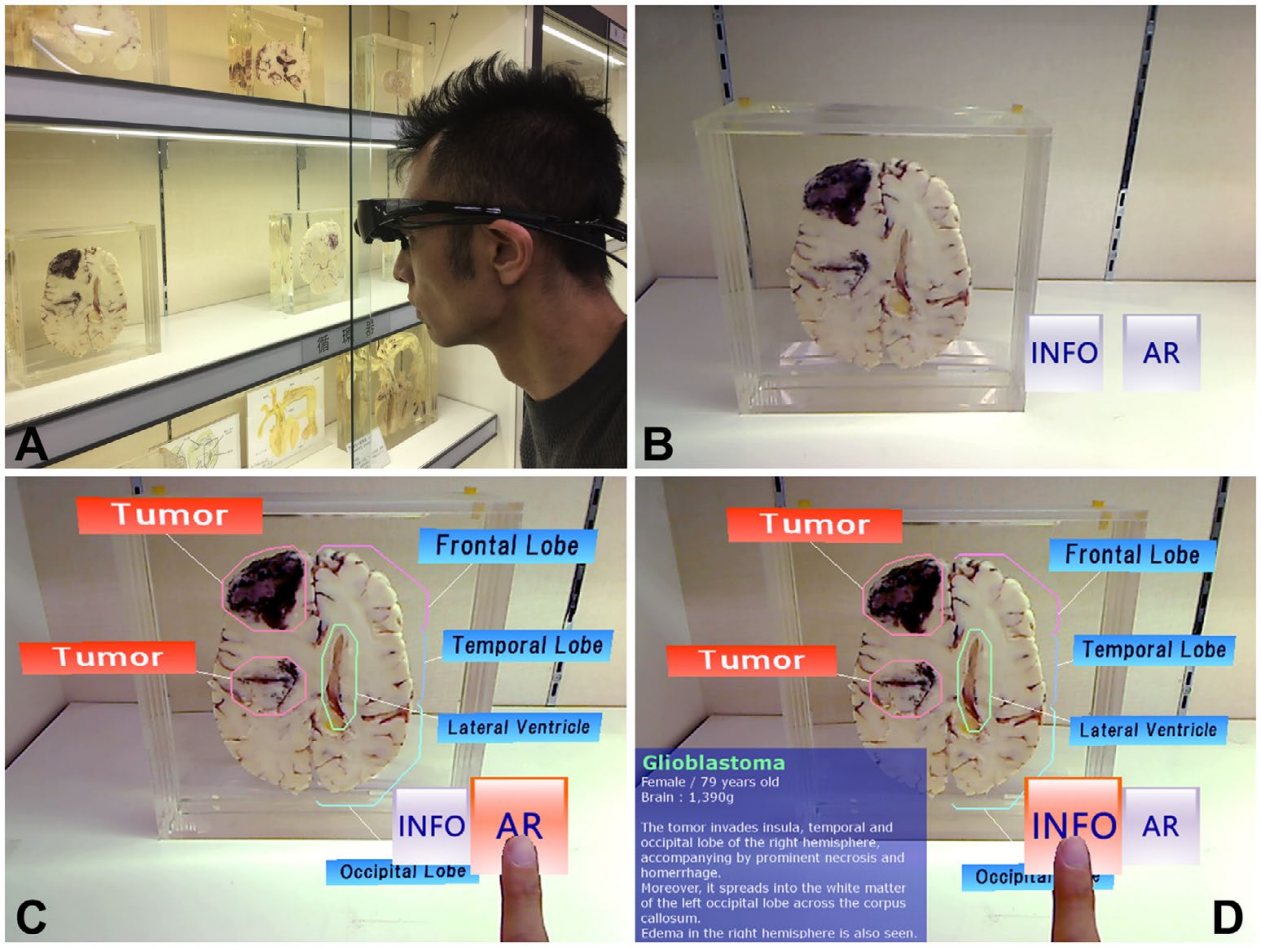
A head‐mounted display augmented reality (HMD AR) system with a natural click interface. A, A user capturing the cross section of a specimen with the HMD’s camera; B, Virtual buttons with the display selection buttons added at the corner of the display area on the HMD display; C, Part labels displayed on the specimen when the user operates the AR button with a click gesture. These are not displayed unless the user pulls them up again D, Information label of the specimen displayed when the user operates the information (INFO) button. This is not displayed unless the user pulls it up again.

This study set two specific objectives to realize its aim: (1) to optimize the AR techniques with which a real specimen in the museum is viewed to establish a new exhibition description and (2) to compare two AR systems alongside traditional description panels, with an additional learning experience evaluation. In each process, the present study prioritized the results from a subjective evaluation through questionnaire surveys in order to focus on user‐centered design. The first objective, which is the optimization of the AR techniques, included two‐step usability evaluations of AR tracking techniques (i.e., AR marker and object image marker systems) and display devices (i.e., tablet AR and head‐mounted display AR) via a pilot study with questionnaire surveys. The second objective, which is the comparison of two AR systems alongside traditional description panels, also involved a pilot study with a questionnaire survey on the perception of AR systems, with the addition of a learning experience evaluation. In the first evaluation of objective 1 (i.e., AR tracking technique evaluation), two systems with different AR tracking techniques were constructed: AR marker and object image marker systems (simply referred to as the image marker system). The AR marker system was developed using ARToolKit, version 2 (ARToolworks Inc., Seattle, WA) and VisualStudio 2013 professional (Microsoft Corp., Redmond, WA) on a desktop computer (ENVY, Hewlett Packard Co., Palo Alto, CA) and a tablet device (Surface Pro 3, Microsoft Corp., Redmond, WA). ARToolKit software is an open‐source library used to construct simple and low‐cost AR environments using AR markers. It enables a developer to easily implement processing steps, namely setting AR marker images, detecting captured images, and presenting information in the AR environment (Figs. 2 A and B). The image marker system was constructed using the same configurations as those for the AR marker, with the exception that it used an updated version of the library software, ARToolKit, version 5 (DAQRI, Los Angeles, CA), because it enables the setting of the object image as an AR marker (Fig. 2). In this system, a rectangular portion of each cross‐section image with characteristic features is clipped from the overall cross‐section image and utilized as an image marker (Fig. 2C). To match the AR information to a traditional specimen display, the following points were taken into consideration: (1) the requisite minimum number of virtual labels and pointing lines with light color were chosen and arranged so as not to obscure the specimen image (Fig. 2); (2) the information label was displayed with an opacity level of 0.5 (where 0 is completely transparent, 0.5 is 50% see‐through, and 1 is not transparent at all) to prevent the specimen image from being concealed from view (Fig. 3).

In the second evaluation (i.e., display device evaluation), a tour support system using a video see‐through head‐mounted display (HMD; Wrap920AR, Vuzix Corp., West Henrietta, NY) was constructed. The HMD presented a display in front of both viewer eyes with a horizontal viewing angle of 31° and was connected to a laptop computer (Think Pad, Lenovo Corp., Hong Kong, China). Although the HMD device does not require users to hold the tablet toward each exhibit, it cannot perform touch‐screen operations. A more significant disadvantage is that it requires the operation of an additional computer keyboard connected to the HMD. A natural click interface technique (Sugiura et al., 2014) was incorporated into the HMD system to avoid this inconvenience. The technique recognizes users’ hand movements, the so‐called “click gestures,” and operates the virtual object. A click gesture refers to the pressing of a button with a finger in the real world and pointing to a virtual object with a finger in the virtual world. With this technique, users can intuitively operate a virtual object (button) with a simple click gesture for displaying virtual labels or information (Fig. 3). In case users do not require such AR explanations or prefer not to have them, virtual labels are not displayed they are brought up by the user.

A questionnaire survey was conducted to provide a subjective evaluation on the usability of each system. All surveys were approved by the Research Ethics Committee of the Faculty of Medicine, University of Yamanashi. Enrollment was voluntary, and signed consent forms were obtained from all participants. Study participants were monitored for any symptoms associated with cybersickness (which consists of symptoms similar to motion sickness and eye strain) while using AR technology (Rebenitsch and Owen, 2016). A total of 60 participants studying anatomy for the first year in a nursing course and the second year in a medical course (24 males and 36 females with a mean age of 19.4 ± 2.0 years), and another 60 participants studying anatomy from the first‐ and second‐year medical courses (38 males and 22 females with a mean age of 21.6 ± 3.5 years) were recruited as participants to evaluate the AR tracking and display devices, respectively. All participants reported having no experience using systems with either a tablet AR or an HMD AR. Following instructions on how to use the two AR systems, each participant freely visited two different exhibition booths, each comprised of 10 specimens, with either an AR marker or an image marker. The order of visitation to the two booths was changed randomly for every participant. The participants answered either yes or no to each of the three questions regarding usefulness, ease, and appearance (AR tracking evaluation)/burden of device (display device evaluation). Participants were also able to provide free text comments in the survey as well. All scores were expressed as percentages. Data were analyzed by the chi‐squared test using the free statistical software R, version 3.4.3 (R Development Core Team, Vienna, Austria).

The second objective (i.e., comparative evaluation of the two AR systems and the traditional method) aimed to compare the developed AR systems with the traditional methods for exhibition description from two viewpoints: effectiveness of learning (achievement test; see Supplementary Material for examples of questions asked) and a questionnaire survey on AR perception. A total of 84 participants studying anatomy from the first‐ and second‐year medical courses (52 males and 32 females with a mean age of 20.8 ± 2.5 years) were recruited. The participants were divided into three sample groups: control (traditional exhibition description with no AR support), tablet AR, and HMD AR. The first two groups each consisted of 32 participants, and the third group had 20 participants. The participants in the groups with the AR support experienced either of the two previous usability studies in order to be well accustomed with the device, and to simply compare the learning achievement in the three groups. The total time spent observing the specimens was measured from the video recording data using a portable device (iPad Pro, Apple Inc., Cupertino, CA). Each participant freely visited eight pathological specimens arranged in three adjacent exhibition booths. A paper‐based pathology knowledge test consisting of eight questions with five choice answers was used to measure the learning effectiveness of the experiences (Appendix). Subsequently, the participants were asked to complete a questionnaire on their perception of the AR experience, their level of learning, and their satisfaction. The questionnaire was comprised of 10 statements for three themes (Table 2), with a five‐point Likert scale from strongly disagree (score 1) to strongly agree (score 5). The reliability of the questionnaire was evaluated by the Cronbach’s alpha internal consistency test. The coefficient for the entire questionnaire was 0.889, and those for each questionnaire theme were 0.787, 0.765, and 0.750 for AR experience, level of learning, and satisfaction, respectively, suggesting that their reliability was acceptable. The data were statistically evaluated through the Kruskal–Wallis test and the Wilcoxon rank‐sum test with Bonferroni correction for multiple comparisons using the R software, version 3.4.3 (R Development Core Team, Vienna, Austria).

**Table 1:** Survey Responses Regarding Usability of the Augmented Reality System in Medical Specimen Museum Tours

Statement	AR tracking technique			Display device		
	AR marker Yes, N (%)	Image marker Yes, N (%)	Chi‐square *P*‐value	Tablet AR Yes, N (%)	HMD AR Yes, N (%)	Chi‐square *P*‐value
1. I think that this system is overall useful	60 (100.0)	60 (100.0)	—	60 (100.0)	60 (100.0)	—
2. It was easy to access information about the exhibits	19 (31.7)	42 (70.0)	<0.001^a^	46 (76.7)	45 (75.0)	0.830
3. I think that the exhibition appearance is favorable (AR tracking technique)	18 (30.0)	60 (100.0)	<0.001^a^			
4. The device was tiring to use (display device)				41 (68.3)	15 (25.0)	<0.001^a^

a *P* < 0.05, Chi‐squared test; Usability evaluation of AR tracking technique for the AR marker and the image marker systems (*N* = 60); Usability evaluation of the display device for the tablet AR and the HMD AR (*N* = 60); AR, augmented reality; HMD, head‐mounted display.

**Table 2:** Results of the Questionnaire Survey Regarding Experience, Learning, and Satisfaction with Using Augmented Reality System in Medical Specimen Museum Tours.

Theme/Statement	Control Mean (±SD)	Tablet AR Mean (±SD)	HMD AR Mean (±SD)	Control vs. Tablet AR *P*‐value	Control vs. HMD AR *P*‐value	Tablet AR vs. HMD AR *P*‐value
Augmented reality experience	3.75 (±0.89)	4.24 (±0.71)	4.23 (±0.61)	<0.001^a^	<0.001^a^	1.000
1. The exhibition description is useful	3.66 (±0.81)	4.09 (±0.63)	4.25 (±0.62)	0.061	0.027^a^	1.000
2. The quality of the description helped to hold my attention	3.66 (±0.99)	4.47 (±0.66)	4.30 (±0.64)	0.002^a^	0.064	0.961
3. Motivates learning	3.50 (±0.90)	4.00 (±0.79)	4.05 (±0.59)	0.120	0.080	1.000
4. The exhibition description is helpful for learning	4.00 (±0.75)	4.41 (±0.66)	4.30 (±0.56)	0.088	0.505	1.000
Level of learning	3.75 (±0.89)	4.23 (±0.78)	4.18 (±0.62)	<0.001^a^	0.010^a^	1.000
5. The description approach is understandable and familiar to me	3.56 (±0.97)	4.22 (±0.78)	4.05 (±0.67)	0.025^a^	0.260	1.000
6. It is easy to understand the characteristics of diseases via the description	3.69 (±0.77)	4.16 (±0.83)	4.25 (±0.43)	0.101	0.017^a^	1.000
7. It is an effective learning tool	4.00 (±0.87)	4.31 (±0.73)	4.25 (±0.70)	0.480	1.000	1.000
Satisfaction	3.63 (±0.85)	4.25 (±0.72)	4.27 (±0.63)	<0.001^a^	<0.001^a^	1.000
8. I enjoyed learning with the descriptions	3.63 (±0.89)	4.28 (±0.76)	4.35 (±0.73)	0.013	0.017^a^	1.000
9. The overall assessment of the description is positive	3.66 (±0.85)	4.38 (±0.65)	4.25 (±0.54)	0.002^a^	0.032^a^	1.000
10. The tour using the description gave me a satisfying feeling of accomplishment	3.59 (±0.79)	4.09 (±0.72)	4.20 (±0.60)	0.032^a^	0.021^a^	1.000

a *P* < 0.05, Wilcoxon rank‐sum test with Bonferroni correction; Mean scores and ±standard deviations (SD) are based on a five‐point Likert scale (1 = strongly disagree and 5 = strongly agree); Number of participants: control group (*N* = 32), tablet AR group (*N* = 32), and HMD AR group (*N* = 20); AR, Augmented reality; HMD, Head‐mounted display.

## Results

### Optimization of the Augmented Reality Technique (Objective 1)

In the AR tracking evaluation, all of the participants responded to the question (Q1) whether each system was useful (Table 1). Some of the comments in free text were as follows: “*I was attracted by the specimens through the AR guide*” or “*The AR information facilitated my understanding of the diseases*.” The AR system itself seemed to have been favorably accepted as a useful tool.

Second, regarding ease (Q2), 31.7% (19 out of 60) of the participants responded “yes” for the AR marker system and 70.0% (42 out of 60) for the image marker system. The difference in the rate of positive responses was statistically significant (χ^2^ = 17.64, *P* < 0.001), suggesting the superiority of the image marker system. Furthermore, the participants who responded “no” (68.3% (41/60) and 30.0% (18/60) for the AR marker and image marker, respectively) stated the following in their free comments: “*The information of the AR marker system was not displayed when only the tablet’s camera captured each specimen image*,” “*The wait time for displaying information was somewhat frustrating in the image marker system*,” and “*Regardless of the system, holding the tablet became increasingly difficult (because of its weight) while observing each specimen*.”

Third, in terms of the appearance question (Q3), all of the participants had favorable opinions of the image marker system, whereas only 30.0% (18/60) of the participants had positive opinions regarding the AR marker system. The difference was statistically significant (χ^2^ = 59.53, *P* < 0.001). The large difference suggested the clear superiority of the image marker in appearance to the AR marker. The free comments of those who responded unfavorably were as follows: “*AR markers appear to intrude on the viewing of the exhibits*” or “*I think AR markers disfigure the overall exhibition*.”

The results of the pilot study suggested that the image markers would provide a more favorable impression to the participants than the AR markers. On the basis of these results, the image marker system was adopted as the system for the AR tracking technique. Given the comment that holding the tablet was a burden to users, a system using the HMD instead of the tablet was developed, and a second pilot study was performed to evaluate the usability of the display device.

In the usefulness question (Q1) of the display device evaluation, all of the participants responded that both systems were useful. The AR‐based system was useful to users despite the different devices. Regarding ease (Q2), 76.7% (46/60) of the participants for the tablet AR and 75.0% (45/60) for the HMD AR responded that access to information was easy, respectively. No significant difference was found between them (χ^2^ = 0.05, *P* = 0.830). Several comments described some irritation with the wait time before display of AR information. Approximately one‐fourth of the participants (23.3% for tablet AR and 25% for HMD AR) disagreed, which could partially be due to this issue. Regarding device burden (Q3), the number of participants who agreed that the device was tiring to use was 68.3% (41/60) for the tablet AR and 25.0% (15/60) for HMD AR. The difference was statistically significant (χ^2^ = 22.63, *P* < 0.001). Some of the comments on the positive or negative points in the free descriptions were are as follows: “*I was comfortable with HMD because it was a completely hands‐free device, whereas I got tired of holding the tablet*,” “*I had some difficulty in viewing with the HMD display because of the small screen size and the low image resolution,*” and “*Continuing this visit for a longer period would induce dizziness with either of the device tools*.”

These results suggest that (1) AR‐based systems are favorable to students regardless of the device and that (2) HMD avoids the physical inconvenience of carrying a tablet device, but may have disadvantages, such as poor visibility due to the small display size with low resolution.

### Comparison of Two Augmented Reality Systems with Traditional Exhibit Descriptions (Objective 2)

**Figure 4:**
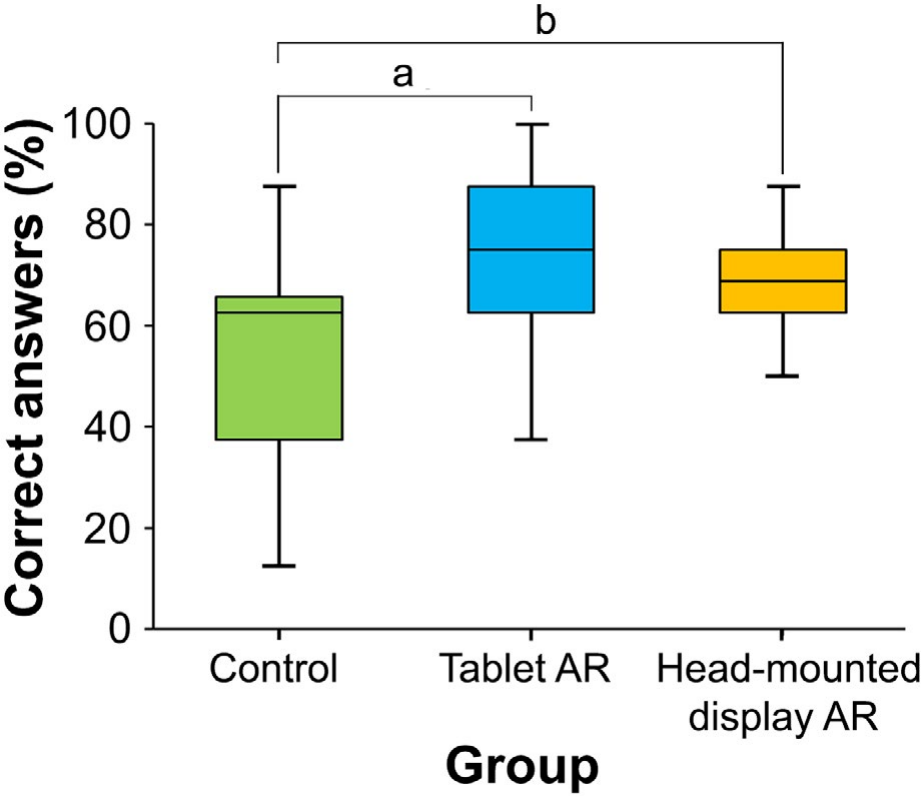
Box‐and‐whisker plot of the overall mean scores of correct answers (%) obtained in the pathological knowledge test. Control group (*n* = 32), tablet augmented reality (Tablet AR) group (*n* = 32), and head‐mounted display AR group (*n* = 20); ^a^ *P* < 0.001; ^b^ *P* = 0.018 (Wilcoxon rank‐sum test with Bonferroni correction).

The median placement accuracies of the total achievement test scores for the control, tablet AR, and HMD AR were 62.5%, 75.0%, and 68.8%, respectively (Fig. 4). Significant differences were found among the three groups (χ^2^ = 19.95, *P *< 0.001). The Wilcoxon multiple comparison showed that the total scores for both AR groups were significantly higher than that of the control group (*P* < 0.001 and *P* = 0.018 for control vs. tablet AR and control vs. HMD AR, respectively), whereas no significant difference was found in the scores between the two AR groups (*P* = 0.247). These results suggest that both AR groups could improve learning achievements regardless of the difference in the device. The mean values (±SD) of the total time spent were 3 minutes: 15 seconds (±1:23), 5 minutes: 01 second (±1:27), and 4 minutes: 25 seconds (±0:47) for the control, tablet AR, and HMD AR, respectively. A significant difference was found in the total time spent for the three groups (Kruskal–Wallis test, χ^2^ = 18.96, *P* < 0.001). The Wilcoxon rank‐sum test with Bonferroni correction showed that the total time spent for both AR groups was significantly longer than that for the control (*P* < 0.001, *P* = 0.008 for the control vs. tablet AR and control vs. HMD AR groups, respectively). Use of the AR device was likely to extend the time spent observing the exhibition descriptions by increasing participant motivation and/or attention. In the tablet AR case, the time spent tended to be longer than that for the HMD AR group, but no significant difference was found (*P* = 0.518). These results suggest that, regardless of the device, AR information can facilitate learning activity among participants. The following free comments support this assumption: “*I could follow the tour attentively (on the tablet AR),” “I regard the tools as fairly effective for learning (on the tablet AR),” “It was truly an inspiring experience with the new type of description. I would like to have more visits to other specimens as well (on the HMD AR),”* and “*It was not easy to get what the key points were, so I finished my tour immediately (on control).”*

The results from the questionnaire on perception showed that the scores for both AR groups were more than 4.0, whereas most of the scores for the control group were less than 4.0. A significant difference was found among the three groups (χ^2^ = 77.06, *P *< 0.001) (Table 2). The overall mean scores of all themes for both AR groups were significantly higher than that for the control (*P* < 0.001 and *P* < 0.001 for control vs. tablet AR and control vs. HMD AR, respectively). A significant difference was not observed in any pair of items in the comparisons between the tablet AR and the HMD AR. The overall results imply that AR support can facilitate learning activities by increasing students’ attention and/or motivation, and may thus lead to improved learning outcomes.

## Discussion

The University of Yamanashi, Faculty of Medicine specimen museum houses more than 500 specimen collections including tissue specimens. The macroscopic specimens are arranged by systems. The booth of normal structure specimens contained cross sections of the head, thorax, abdomen, and upper/lower limbs, and the whole fetal specimens organized by developmental stages. The specimens of various organ diseases are colored in order to replicate their original state according to the method of the Medical Museum of Kawasaki Medical School (KMS, 2012). This feature provides visitors with a practical visualization of the pathological state of the organs. In essence, these specimens can provide visitors with a valuable opportunity to acquire new knowledge and satisfy their curiosity regarding the macroscopic structure and various diseases of the human body, as well as to engender thoughts regarding the dignity of life and respect for organ donation. However, the traditional style of exhibition descriptions may not be as attractive or fascinating to modern students with experience in multimedia information tools. In the present study, new tour support systems were constructed using AR technology to make the traditional museum more attractive and effective as a learning support environment. Moreover, these systems were evaluated by pilot studies with the participation of medical and nursing course students based on the two specific objectives (objectives 1 and 2). Objective 1 contained two‐step usability evaluations for optimizing AR tracking and display devices. As a first step, usability was evaluated by comparing the use of AR markers against image markers. Based on the results of the pilot study, the image marker was adopted as the optimal method for AR tracking. The second step was to evaluate the usability of a different display device by comparing the tablet AR with the HMD AR. The results indicated that both systems are supported more favorably than the traditional style of exhibition description panels. However, some inconveniences were unveiled, namely the physical inconvenience of holding the tablet, as well as poor visibility due to the small display size and low‐image resolution. From the viewpoint of cognitive or perceptional psychology, Gibson (1979) introduced the term “affordance” that refers to physical and social implications of objects and their relationship to human beings. The term was defined as perceivable action possibilities in the field of the human–computer interactions, indicating that an ideal program design does not require any instructions, because users can guess by a simple look at the objects (Norman, 1988). The two‐step system adjustments in the present study based on usability (i.e., changing the AR markers to image markers and changing of the device from a tablet AR to an HMD AR) rendered the system design more likely to fit human perception, conforming to the concept of affordances. Studies on the affordance of AR in pedagogical application tools have been increasing (Dunleavy et al., 2009; Faiola and Matei, 2010; Cheng and Tsai, 2013; Wu et al., 2013).

Objective 2 aimed to compare two AR systems with the traditional method with regards to the effectiveness of learning (evaluated by achievement test) and perception. Although the number of samples and questions were small, the achieved score was significantly higher for the tablet AR and the HMD AR than for the traditional description panels. The results suggest that the AR‐based systems are not only effective tools for providing exhibition descriptions, but can also contribute to enhancing students’ motivation for learning. The evaluation of other items for the AR experience, level of learning, and satisfaction suggested an overall superiority of the AR systems to the traditional description panels. Meanwhile, no significant difference was found in the evaluation items between the tablet AR and the HMD AR, suggesting that AR information contents might provide a stronger effect than the differences in the device. According to the cognitive theory of multimedia learning or instructional design (Paas et al., 2003; Mayer, 2009), cognitive load is associated with learning performance, and the goal of instructional design should be to reduce extraneous cognitive load to release working memory. Küçük et al. (2016) examined medical students’ academic achievements and cognitive load for anatomy learning. Their results demonstrated a clear reduction in the cognitive load when using mobile AR. Although the present study did not specifically focus on such an evaluation, the results from the survey questions suggested that (1) AR systems could have reduced the cognitive load in comparison with the traditional exhibition description (i.e., with no AR support); (2) the image marker system could have reduced the load more than the AR marker system (Table 1); and (3) the HMD AR could have reduced the load more than the tablet AR (Table 2). Despite the advantage of the AR technique, several negative factors were associated with the physical inconvenience of holding the tablet and poor visibility caused by the display size and resolution, which would be resolved by technological developments in the future. Surveys addressing this issue should be conducted in the future.

An overview of the present study encourages a new approach that not only provides an attractive learning environment for students but also adds value to the discipline of anatomy. The use of AR to complement visits to the anatomical museum could be a revolutionary change for the future of anatomical exhibitions. From the viewpoint of translational research, the AR application design in the present study could be applicable to other medical museums and institutes, and also to lower socioeconomic areas, because it is feasible and cost‐effective, and it can be replicated with ease.

From the curator’s point of view, two issues should be considered. First, in contrast to the usefulness of AR tools, visual display technologies, especially HMD, have been associated with “cybersickness,” which has similar symptoms to motion sickness, resulting in nausea, headaches, and dizziness (Howarth and Costello, 1997; Rebenitsch and Owen, 2016). Moro et al. (2017) reported that one‐third of VR users experienced blurred vision and 21% had double vision. In addition, these symptoms were significantly higher in VR users than in AR and tablet users. In the present study, 1.9 % (3/152) of the participants using the tablet and 10.0 % (8/80) of those using HMD experienced dizziness, although no participants abstained from the experiment despite the fact that their participation was completely voluntary and could be withdrawn at any time. In the future, an optical see‐through HMD with a high display resolution could provide a more comfortable environment without causing blurred vision or dizziness. Second, the curator should carefully consider how the AR tour support system is implemented as a learning tool. Because medical museums house sensitive human remains and images, videos, and photographs are generally not allowed. The tablets and HMD devices used in the present study were prepared by curators for this specific use and were loaned to visitors during tours inside the museum. However, mobile terminals and other image‐recording devices are generally not allowed to prevent visitors from recording videos or taking images of the sensitive material on display.

### Limitations and Further Studies

This study has several limitations. This study examined these modalities’ usability and effectiveness for learning, and surveys were conducted as pilot studies with a limited number of medical, nursing, and paramedical course students. A greater number of participants with a wider range from novice students to residents or experienced professionals are necessary to confirm the present conclusion. Additionally, the difficulty level of examination questions must be varied depending on the experience of participants. The same can be said for the contents of AR information that could be varied with each participant’s proficiency level or interest. The following requests were indicated in the free text comments from the participants in the survey: “The system would be better if it could show more AR contents,” “It would be easier to understand if some CG contents would be shown,” and “I’m hoping it would display AR information related to clinical nursing.” The content of the information can be extended or improved through the following approaches: (1) additional labels for supplemental explanation such as anatomical 3DCG showing the 3D structural relation within the whole body or in relation to the vascular or the surrounding skeletomuscular system; (2) a smart system that can propose appropriate sets of AR information by estimating each visitor’s level or interest based on an analysis of their operations and that can even select optimum tour routes; and (3) hands‐on operations with gestures other than the natural click, such as sliding, pinching, and grasping. These approaches would facilitate active operating behavior for a more effective learning, thus providing an active learning framework.

Another limitation is the financial matter of using mobile or wearable devices. A sufficient number of devices cannot be prepared at one time, especially in the case of school visits as a course curriculum, because all devices are prepared by curators. Application software that can be installed on each visitor’s mobile terminal would be a solution if strict conditions were secured for use exclusively during the tour without recording images. A low‐cost optical see‐through HMD with a high display resolution will be a better solution for avoiding symptoms of cybersickness in the future.

## Conclusions

This study developed AR‐based support systems to establish new learning modalities for tours in a medical specimen museum. To the best of the authors’ knowledge, this study is the first to report of a system that uses the specimen image as an image marker and employs a natural click interface for tours in a specimen museum. Based on the pilot survey results, the system was well optimized as a useful tool within the constraints of user‐centered design. The learning effectiveness of the AR‐based system was also confirmed. In conclusion, AR‐based systems not only can be effective tools for exhibition descriptions but can also contribute to enhancing students’ motivation for learning. The application of the AR technology within the anatomical museum may even provide a paradigm shift that could be expanded throughout the world, with AR‐based systems shaping the future of anatomical exhibitions. An HMD AR system with a natural click interface seems to be highly scalable in terms of not only the button arrangement but also other display contents. Further improvement of the system is expected to enhance the learning efficiency of anatomical education.

## Notes on Contributors

ATSUSHI SUGIURA, Ph.D., is an assistant professor in the Interdisciplinary Graduate School at the University of Yamanashi, Chuo, Yamanashi, Japan. He teaches experimental medicine and physics and his research interests include augmented reality and computer and human vision.

TOSHIHIRO KITAMA, Ph.D., is an associate professor at the Interdisciplinary Graduate School at the University of Yamanashi, Chuo, Yamanashi, Japan. He teaches physiology, anatomy, and experimental medicine, and his research interests include oculomotor and posture control in systems neuroscience. He is a member of the Society for Neuroscience (SfN).

MASAHIRO TOYOURA, Ph.D., is an associate professor in the Interdisciplinary Graduate School, at the University of Yamanashi, Kofu, Yamanashi Japan. He teaches computer graphics and affective computing, and his research interests include augmented reality and computer and human vision. He is a member of the Association for Computing Machinery Special Interest Group on Computer Graphics and Interactive Techniques (ACM SIGRRAPH) and the Institute of Electrical and Electronics Engineers (IEEE) Computer Society.

XIAOYANG MAO, Ph.D. is a professor at the Interdisciplinary Graduate School, University of Yamanashi, Kofu, Yamanashi Japan. She teaches computer visualization and discrete mathematics, and her research interests include texture synthesis and non‐photo‐realistic rendering and their applications to scientific visualization. She is a member of the Association for Computing Machinery Special Interest Group on Computer Graphics and Interactive Techniques (ACM SIGRRAPH) and the Institute of Electrical and Electronics Engineers (IEEE) Computer Society.
