# Supplementary material for: The Use of Augmented Reality Technology in Medical Specimen Museum Tours
Source: Anat Sci Educ. 2018 Nov 19;12(5):561–71. doi: 10.1002/ase.1822 (PMC6767574; doi:10.1002/ase.1822)
Supplement: Supplementary file 1 [file ASE-12-561-s001.docx]

Appendix.

Examples of questions for the pathology knowledge test with five choice answers.

| Question |  | Choices |
| --- | --- | --- |
| 1. What part of the brain is often affected by |  | A. Cerebellum |
| the Glioblastoma? |  | **B. Cerebrum** |
|  |  | C. Spinal cord |
|  |  | D. Brain stem |
|  |  | E. Hypothalamus |
| 2. What is the main cause of myocardial |  | A. Depressed cardiac function |
| infarction? |  | **B. Atherosclerosis in the coronary arteries** |
|  |  | C. Anomalous origin of the coronary artery |
|  |  | D. Cardiac valve dysfunction |
|  |  | E. Arrhythmia |
| 3. What sort of symptom can be observed |  | **A. Fibrosis (growth of fibrous tissue)** |
| within the pulmonary interstitium in the case of |  | B. Calcification |
| interstitial pneumonia? |  | C. Re-epithelialization |
|  |  | D. Edema |
|  |  | E. Canceration (growing into a cancer) |
| 4. What part of the lung is often affected by |  | A. Pulmonary vascular |
| the squamous cell carcinoma? |  | **B. Hilar bronchi** |
|  |  | C. Bronchi of the lung field |
|  |  | D. Pleura |
|  |  | E. Apex of the lung |
